# Rosmarinic Acid Alleviates Cisplatin‐Induced Acute Kidney Injury by Targeting FYN to Regulate Ferroptosis

**DOI:** 10.1002/fsn3.72281

**Published:** 2026-08-27

**Authors:** Chan Huang, Huimin Shi, Shuang Xu, Gaoting Qu, Ningxun Cui, Lu Jiang, Aiqing Zhang, Wei Zhou

**Affiliations:** ^1^ Department of Nephrology and Immunology Children's Hospital of Soochow University Suzhou Jiangsu People's Republic of China; ^2^ Department of Nephrology, National Clinical Research Center for Children and Adolescents' Health and Diseases, Children's Hospital Zhejiang University School of Medicine Zhejiang Hangzhou People's Republic of China; ^3^ Department of Pediatrics The Second Affiliated Hospital of Nanjing Medical University Nanjing Jiangsu People's Republic of China; ^4^ Department of Pediatrics The First Affiliated Hospital of Henan University of Chinese Medicine Zhengzhou Henan People's Republic of China

**Keywords:** CDDP‐AKI, ferroptosis, FYN, rosmarinic acid

## Abstract

Cisplatin (CDDP) is a widely used chemotherapeutic agent, but its nephrotoxic side effects limit its clinical application, which can lead to severe acute kidney injury (AKI). Growing evidence indicates that ferroptosis plays a critical role in CDDP‐AKI. Rosmarinic acid (RA) is a natural polyphenolic compound extracted from various plants that has been demonstrated to possess significant antioxidant and anti‐inflammatory activities. This study aims to investigate whether RA protects against CDDP‐AKI by regulating ferroptosis. We demonstrated that RA significantly ameliorated renal dysfunction, pathological damage, and inflammatory responses in CDDP‐AKI mice. Furthermore, RA effectively suppressed ferroptosis by regulating the Nrf2/HO‐1 signaling axis, as evidenced by reduced lipid peroxidation levels and restored expression of key ferroptosis‐related proteins (GPX4 and xCT). Integrating network pharmacology predictions with molecular docking, we identified FYN as a potential direct target of RA and further verified that RA inhibits the expression and activation of FYN. Additionally, pharmacological inhibition of FYN using Saracatinib in vivo or genetic knockdown of FYN in HK‐2 cells effectively alleviated CDDP‐induced renal tubular injury and lipid metabolic dysregulation. In conclusion, our findings indicate that RA alleviates CDDP‐AKI by targeting FYN to regulate the ferroptosis pathway, highlighting that RA is a promising candidate for preventing and treating CDDP‐AKI.

## Graphical abstract

Rosmarinic acid alleviates cisplatin‐induced kidney injury by inhibiting FYN phosphorylation and promoting Nrf2 nuclear translocation, which restores GPX4 expression, limits lipid peroxidation, and suppresses ferroptosis in renal tubular epithelial cells.

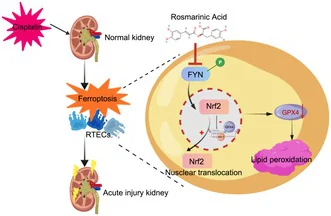

## 1 Introduction

Acute kidney injury (AKI) is a critical clinical syndrome characterized by a rapid decline in renal function, arising from multifactorial etiologies such as drug toxicity, ischemia–reperfusion injury, and infections (Kellum et al. 2021). Cisplatin (CDDP) is a widely utilized chemotherapeutic agent and is employed as a therapeutic agent because of its efficient cytotoxic activity for various solid malignancies, such as lung cancer (Xuzhang et al. 2024), bladder cancer (Jiang et al. 2021), and ovarian cancer (Shao et al. 2023). However, its clinical application is significantly limited by dose‐dependent nephrotoxicity. It is estimated that the incidence of AKI in patients treated with CDDP is up to 30%, significantly increasing mortality rates and often forcing the discontinuation of CDDP treatment due to the absence of effective therapies for AKI (Sears and Siskind 2021).

CDDP‐AKI is characterized by proximal tubular epithelial cell damage, traditionally attributed to mechanisms involving oxidative stress, mitochondrial dysfunction, and activation of apoptotic pathways (Tang et al. 2023). However, emerging evidence highlights a critical role of ferroptosis, a novel iron‐dependent form of regulated cell death driven by lipid peroxidation in AKI resulting from diverse etiologies (Feng et al. 2022). Ferroptosis is driven by the accumulation of iron‐catalyzed lipid peroxides, which disrupts membrane integrity and ultimately leads to cell death (Dixon and Olzmann 2024). CDDP exacerbates this pathological process by aggravating intracellular iron overload, amplifying the lipid peroxidation cascade, leading to glutathione depletion, and inactivating glutathione peroxidase 4 (GPX4) (Wang et al. 2023). The nuclear factor erythroid 2‐related factor 2/heme oxygenase‐1 (Nrf2/HO‐1) signaling pathway exerts a pivotal regulatory effect on the ferroptotic process (Wang, Yu, et al. 2024). Nrf2 is an important antioxidant transcriptional regulatory factor. Upon activation, it translocates into the nucleus and binds to antioxidant response elements (AREs) in the promoter regions of downstream genes, thereby upregulating the transcription of antioxidant genes including HO‐1. This process promotes the synthesis and homeostasis maintenance of glutathione (GSH), inhibits the generation and accumulation of reactive oxygen species (ROS), and consequently blocks the characteristic lipid peroxidation reactions associated with ferroptosis (Li et al. 2020). It has been reported that silybinin inhibits ferroptosis and improves AKI induced by ischemia reperfusion by disrupting the ferritin autophagy process (Deng et al. 2024). In addition, the farnesoid X receptor prevents CDDP‐induced AKI by regulating the transcription of ferroptosis‐related genes (Kim et al. 2022). Based on these results, targeting ferroptosis may become a promising therapeutic approach for CDDP‐AKI.

Rosmarinic acid (RA) is a naturally occurring polyphenolic compound that is widely distributed in medicinal plants of the Lamiaceae family and Boraginaceae family (Ijaz et al. 2023). It has attracted much attention due to its remarkable antioxidant, anti‐inflammatory, and anti‐microbial properties. With the in‐depth research on natural products, the potential therapeutic effects of RA in the fields of chronic inflammatory diseases (Mohmad Saberi and Chua 2023), neurodegenerative diseases (Yang et al. 2023), allergic asthma (Guo et al. 2024), and cancer (Liu et al. 2024) have gradually been revealed. The latest research reports that RA improves various disease conditions by regulating ferroptosis, such as crush syndrome‐related AKI (Qiao et al. 2024), ischemic stroke (Jia et al. 2024), and septic‐related acute respiratory distress syndrome (Zeng et al. 2024). However, the effect of RA on ferroptosis in CDDP‐AKI remains unknown. In this study, we confirmed the potent inhibitory effect of RA on ferroptosis and demonstrated that RA is a potential therapeutic agent for CDDP‐AKI.

## 2 Materials and Methods

### 2.1 Drugs

CDDP (purity: 99.30%, HY‐17394, MCE, Shanghai, China) was dissolved in sterile saline. The ferroptosis inhibitor Ferrostatin‐1 (Fer‐1) (HY‐100579), RA (purity: 99.73%, HY‐N0529), the ferroptosis inducer erastin (HY‐15763), and the FYN inhibitor Saracatinib (HY‐10234) were obtained from MCE (Shanghai, China) and were dissolved in a vehicle solution consisting of 10% DMSO and 90% (20% SBE‐β‐CD in saline).

### 2.2 Animal Treatment

Eight‐week‐old male C57BL/6J mice (weighing 22–24 g) were procured from the Animal Experimental Center of Soochow University. When investigating the role of RA in CDDP‐AKI, the mice were randomly divided into six groups (*n* = 6 per group): control group, CDDP group (single intraperitoneal injection of 20 mg/kg CDDP; Kim et al. 2022; Xiang et al. 2022), CDDP + Fer‐1 group (a single intraperitoneal injection of 5 mg/kg Fer‐1), CDDP + RA‐L group (intraperitoneal injections of 25 mg/kg RA for three consecutive days), CDDP + RA‐M group (intraperitoneal injections of 50 mg/kg RA for three consecutive days), and CDDP + RA‐H group (intraperitoneal injections of 100 mg/kg RA for three consecutive days). Three graded doses of RA were selected in accordance with our safety experiments and previously published literature. In this study, intraperitoneal administration of RA at a maximum dose of 100 mg/kg did not trigger obvious toxic side effects in mice throughout the experimental period, including body weight loss, renal damage, and abnormal behavioral activities. Moreover, published toxicological data have reported that the median lethal dose (LD_50_) of RA is approximately 561 mg/kg (Ghasemzadeh Rahbardar and Hosseinzadeh 2020), further verifying the safety of the dose range adopted in the present study. When exploring the role of pharmacological inhibition of FYN in CDDP‐AKI, the mice were randomly divided into three groups (*n* = 6 per group): control group, CDDP group (single intraperitoneal injection of 20 mg/kg CDDP), and CDDP + Saracatinib group (intraperitoneal injections of 10 mg/kg Saracatinib for three consecutive days). All mice in each group were euthanized at 72 h after CDDP treatment, and serum and renal tissues were collected for subsequent experiments. All animal experiments were approved by the Animal Ethics Committee of Soochow University (approval no. 2025CS071) and adhered to the guidelines formulated by the Chinese Association for Laboratory Animal Science.

### 2.3 Assessment of Renal Function and Histology

Renal function was evaluated by measuring serum creatinine (Scr) and blood urea nitrogen (BUN) levels using a fully automated biochemical analyzer (Hitachi, LABOSPECT008α, China). For histological examination, renal tissues were fixed in 4% paraformaldehyde to prepare paraffin blocks and sections were cut at a thickness of 4 μm. Subsequently, the sections were stained with hematoxylin and eosin (H&E) as well as periodic acid‐Schiff (PAS) according to standard protocols. The renal injury index was scored based on previous reports (Weidemann et al. 2008). After deparaffinization and antigen retrieval of the paraffin sections, IHC or immunofluorescence (IF) staining was performed. The expression levels of target proteins were quantitatively analyzed in terms of the intensity and area of positive staining using Image J software.

### 2.4 Transmission Electron Microscopy (TEM)

Ultrastructural examination of mitochondrial damage in renal tubules was performed by TEM. Briefly, renal tissue specimens of less than 1 mm^3^ were immediately fixed in pre‐cooled 2.5% glutaraldehyde. Following dehydration with a graded acetone series, the samples were embedded in resin blocks. Ultrathin sections (50 – 80 nm) were prepared using an ultramicrotome, followed by dual staining with uranyl acetate and lead citrate. Finally, the sections were imaged under TEM for ultrastructural analysis.

### 2.5 TdT‐Mediated dUTP Nick‐End Labeling (TUNEL) Assay

Renal tubular epithelial cell apoptosis was detected using a Colorimetric TUNEL Detection Kit (C1091, Beyotime, Shanghai, China). Briefly, paraffin‐embedded sections were subjected to dewaxing and rehydration, followed by digestion with Proteinase K and incubation with 3% hydrogen peroxide at room temperature for 15 min to block endogenous peroxidase activity. Subsequently, the TUNEL reaction mixture (comprising TdT enzyme and biotin‐labeled dUTP) was added, and the sections were incubated in a humidified chamber at 37°C in the dark for 60 min. DAB chromogen was then added until brown positive signals became visible. The apoptosis index was calculated using ImageJ software.

### 2.6 Measurement of GSH, MDA and SOD

The levels of GSH, malondialdehyde (MDA), and superoxide dismutase (SOD) in renal tissues or cells were determined using specific commercial assay kits (GSH: KTB1600; MDA: KTB1050; SOD: KTB1030; Abbkine, Wuhan, China) following the manufacturers' instructions. These measurements serve as key indicators of the antioxidant defense capacity and lipid peroxidation levels in tissues or cells. Briefly, renal tissues or cells were homogenized in cold phosphate buffer, and the supernatants were collected by centrifugation for subsequent analysis. All assays were performed using 96‐well plates, and absorbance was measured with an automated Bio‐Rad microplate reader.

### 2.7 Cell Culture and Treatment

HK‐2 cells were purchased from Procell Life Science & Technology Co. Ltd. (Wuhan, China). The cells were maintained in DMEM/F12 medium supplemented with 10% FBS and 1% penicillin–streptomycin solution, and cultured in an incubator at 37°C with 5% CO₂. For drug treatments, cells were pretreated with 20 μM RA or 10 μM Fer‐1 for 1 h, followed by exposure to 10 μM CDDP or 10 μM erastin for 24 h. For lentiviral transduction, short hairpin RNA (shRNA) targeting human FYN was synthesized by GeneCreate Co. Ltd. (Nanjing, China). Transfection was performed using 10 μL of lentiviral particles in the presence of Polybrene per well of a 6‐well plate.

### 2.8 qPCR

Total RNA was extracted from kidney tissues and cells using a Total RNA isolation kit (Vazyme, Nanjing, China). Reverse transcription was performed with the HiScript II 1st Strand cDNA Synthesis Kit (Vazyme, Nanjing, China), followed by PCR amplification using ChamQ Universal SYBR qPCR Master Mix (Vazyme). β‐actin was used as the internal reference, and relative gene expression was calculated using the $2^{-\Delta \Delta Ct}$ method. Sequence information for all primers is provided in Table S1.

### 2.9 Western Blot (WB)

Total proteins were extracted from renal tissues and cells using RIPA lysis buffer (P0013B, Beyotime, Shanghai, China). Cytoplasmic and nuclear proteins were separated using a Nuclear and Cytoplasmic Protein Extraction Kit (P0027, Beyotime, Shanghai, China). Protein concentrations were determined using a BCA Protein Assay Kit (P0009, Beyotime, Shanghai, China). The proteins were then subjected to electrophoresis on a 4% – 20% SDS‐polyacrylamide gel and subsequently transferred onto a PVDF membrane (Millipore, USA). After blocking with NcmBlot Blocking Buffer (P30500, NCM, Suzhou, China) at room temperature for 20 min, the membrane was incubated with primary antibodies at 4°C overnight. The following day, the membrane was incubated with HRP‐conjugated secondary antibodies at room temperature for 2 h. Chemiluminescence detection was performed using an ECL Chemiluminescent Substrate Kit (Abbkine, Wuhan, China) on a chemiluminescence detection system (Bio‐Rad, CA, USA). Quantitative analysis of the protein bands was conducted using ImageJ software. Detailed information on the antibodies used for Western blotting is provided in Table S2.

### 2.10 Cell Viability

Cell viability was assessed using the Cell Counting Kit‐8 (CCK‐8, C0037, Beyotime, Shanghai, China). After the treatment, cells seeded in 96‐well plates were incubated with 10 μL of CCK‐8 solution per well for 2 h. The absorbance was then measured at a wavelength of 450 nm using a microplate reader.

### 2.11 Lipid ROS Detection

Intracellular lipid peroxidation was measured using the Lipid Peroxidation Assay Kit with BODIPY 581/591 C11 (S0043S, Beyotime, Shanghai, China). Cells seeded in 12‐well plates were incubated with 500 μL of the BODIPY 581/591 C11 working solution per well at 37°C for 30 min in a cell culture incubator. After incubation, the cells were washed twice with phosphate‐buffered saline (PBS) and immediately observed under a fluorescence microscope.

### 2.12 Molecular Docking

To investigate the molecular interaction, the three‐dimensional structure of the human FYN kinase domain was acquired from the Protein Data Bank. The structure of RA was downloaded from PubChem. The binding mode and affinity between them were predicted using AutoDock Vina.

### 2.13 Statistical Analysis

All quantitative analyses and outcome assessments of cell experiments (*n* = 3) and in vivo animal (*n* = 6) studies were performed in a blinded condition. All quantitative data are presented as mean ± standard deviation (SD). Normality was assessed using the Shapiro–Wilk test. An unpaired Student's *t*‐test was used for comparisons between two groups, while one‐way analysis of variance (ANOVA) was applied for multi‐group comparisons. Multiple comparisons after ANOVA were conducted using Tukey's post hoc test. All experiments were repeated at least three times independently. *p* value < 0.05 was considered statistically significant. Statistical analyses were performed using GraphPad Prism 10.0 software (San Diego, CA, USA).

## 3 Results

### 3.1 RA Protects Against Renal Dysfunction and Pathological Damage in CDDP‐AKI Mice

**FIGURE 1:**
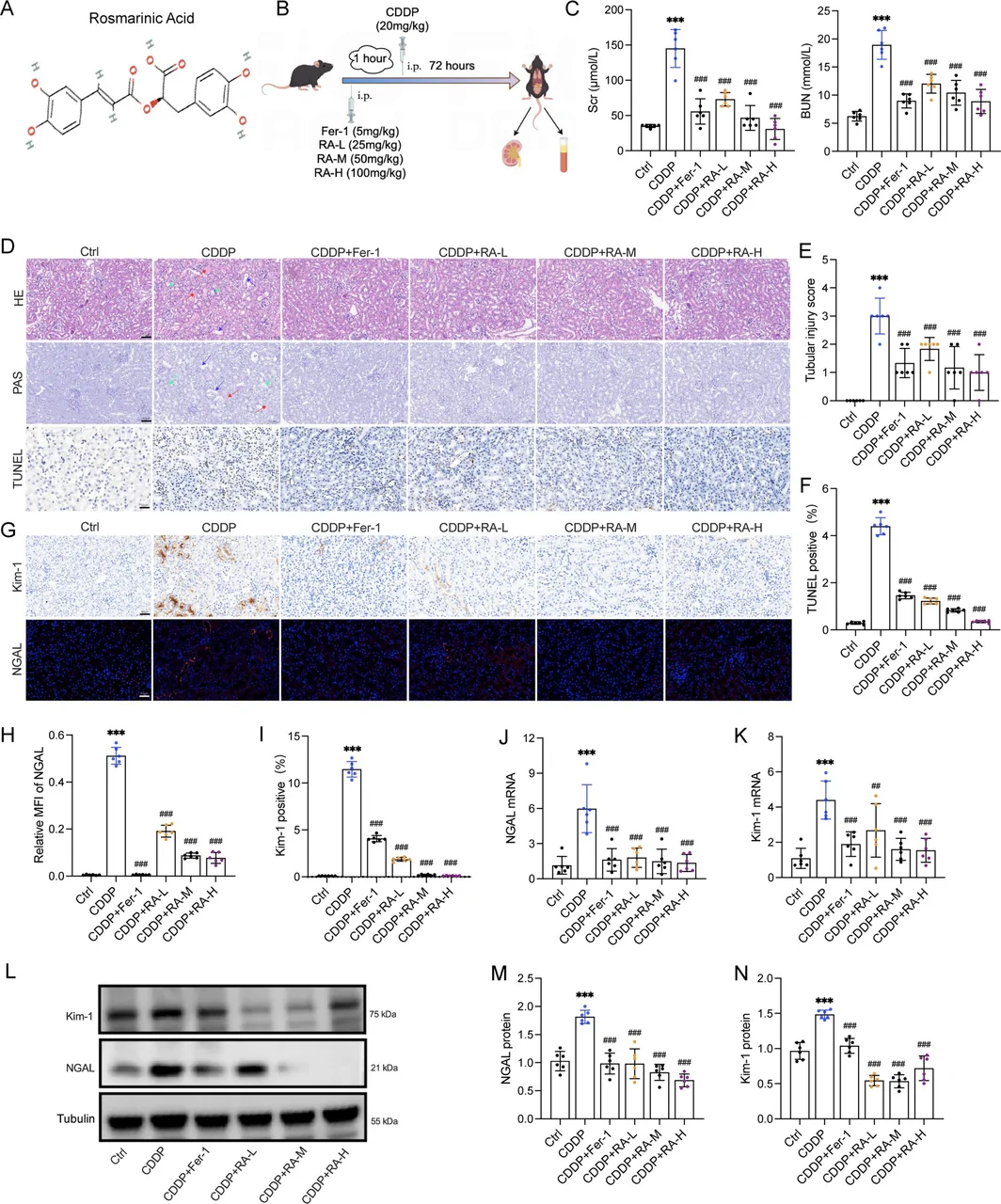
RA ameliorates renal injury in CDDP‐AKI mice. (A) Chemical structure of RA. (B) Schematic diagram of the animal experimental procedure. (C) The levels of Scr and BUN. (D–F) H&E and PAS staining of renal tissues, and tubular injury scores (scale bar = 50 μm, 200× magnification). Red arrows, renal tubular cast formation; blue arrows, renal tubular dilatation and loss of brush border; green arrows, renal tubular necrosis. (G–I) IHC staining for Kim‐1 and IF staining for NGAL in renal tissues, and quantitative analysis (scale bar = 50 μm, 200× magnification). (J, K) Relative mRNA expression levels of NGAL and KIM‐1; (L–N) Protein bands and quantitative analysis of NGAL and KIM‐1. Six biological replicates were included in each group. Data are presented as mean ± SD. Data were assessed by one‐way ANOVA followed by Tukey's multiple comparisons test. ***p < 0.001, versus the control group; ^###^ *p* < 0.001, vs. the CDDP group.

RA (C_18_H_16_O_8_, MW 360.32 g/mol) is an ester composed of caffeic acid and 3,4‐dihydroxyphenyllactic acid (Noor et al. 2022) (Figure 1A). To investigate the pharmacological protective effect of RA on the kidney, this study established a CDDP‐AKI mouse model (Figure 1B). As shown in Figure 1C, the serum levels of Scr and BUN in CDDP‐AKI mice were significantly elevated, indicating obvious renal function impairment. H&E and PAS staining demonstrated that the kidneys of CDDP‐AKI mice exhibited significant tubular injury, characterized by tubular lumen dilation, renal tubular epithelial cell necrosis, protein cast formation, and brush border loss (Figure 1D–F). Furthermore, to clarify the role of ferroptosis in CDDP‐AKI, this study evaluated the intervention effect of Fer‐1. The results showed that Fer‐1 treatment could significantly alleviate renal function impairment and renal pathological damage in CDDP‐AKI mice. Consistent with Fer‐1, RA treatment at different doses all significantly improved the renal function and renal pathological status of CDDP‐AKI mice (Figure 1C–F). IHC and IF results revealed that the protein expression levels of the tubular injury markers Kim‐1 and NGAL were significantly upregulated in CDDP‐AKI mice, while RA treatment remarkably inhibited this trend (Figure 1G–I). qPCR and WB further confirmed that the high expression of KIM‐1 and NGAL at both the mRNA and protein levels induced by CDDP was significantly suppressed after Fer‐1 or RA treatment (Figure 1J–N). These findings indicate that ferroptosis is involved in CDDP‐ AKI, and RA can effectively prevent renal dysfunction and pathological damage in CDDP‐AKI.

### 3.2 RA Alleviates Inflammation in CDDP‐AKI Mice

**FIGURE 2:**
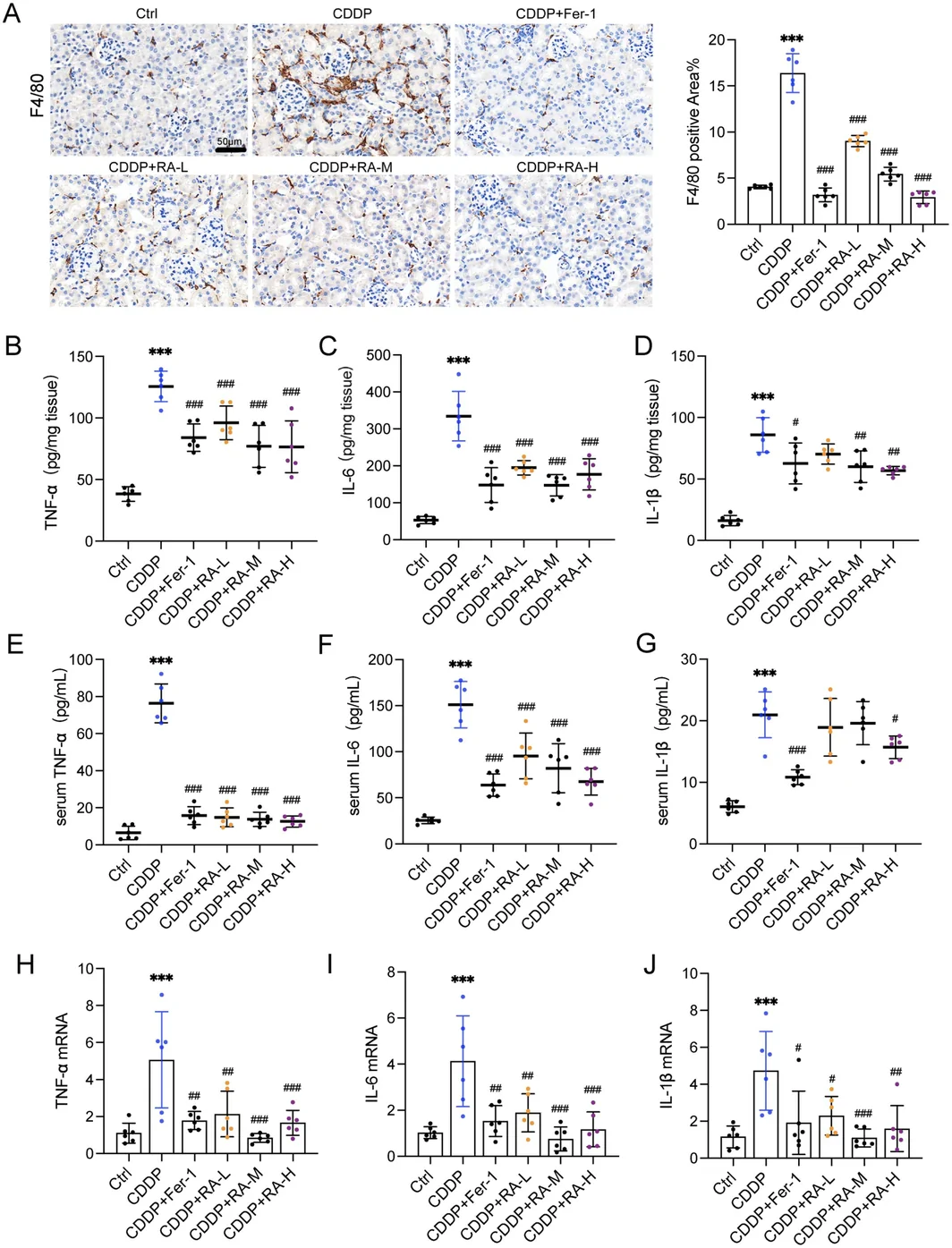
RA ameliorates the inflammatory response in CDDP‐AKI mice. (A) IHC staining for F4/80 expression and quantitative analysis of the positive area (scale bar = 50 μm, 400× magnification). (B–D) ELISA detects the expression levels of TNF‐α, IL‐6, and IL‐1β in renal tissues (E–G) ELISA detects the expression levels of TNF‐α, IL‐6, and IL‐1β in serum. (H–J) Relative mRNA expression levels of TNF‐α, IL‐6, and IL‐1β in renal tissues. Six biological replicates were included in each group. Data are presented as mean ± SD. Data were assessed by one‐way ANOVA followed by Tukey's multiple comparisons test. ***p < 0.001, vs. the control group; ^###^ *p* < 0.001, ^##^ *p* < 0.01, ^#^ *p* < 0.05, vs. the CDDP group.

Immune effector cell infiltration and cytokine storm are important features in the process of CDDP‐AKI (Gao et al. 2024). IHC staining revealed that the F4/80 positive area ratio in renal tissues of CDDP‐AKI mice was significantly larger than that in the control group, while this increase was notably reduced following treatment with Fer‐1 or RA, indicating attenuated macrophage infiltration (Figure 2A). ELISA was performed to determine the effect of RA on the expression levels of inflammatory cytokines in the serum and renal tissues of CDDP‐AKI mice. Compared with the control group, the expression levels of TNF‐α, IL‐6, and IL‐1β were markedly elevated in both the serum and renal tissues of CDDP‐AKI mice, which were reversed by both Fer‐1 and RA treatment (Figure 2B–G). qPCR further confirmed that Fer‐1 and RA significantly suppressed the inflammatory response in the renal tissues of CDDP‐AKI mice, as evidenced by reduced mRNA expression levels of TNF‐α, IL‐6, and IL‐1β (Figure 2H–J).

### 3.3 RA Inhibits Ferroptosis in CDDP‐AKI Mice

**FIGURE 3:**
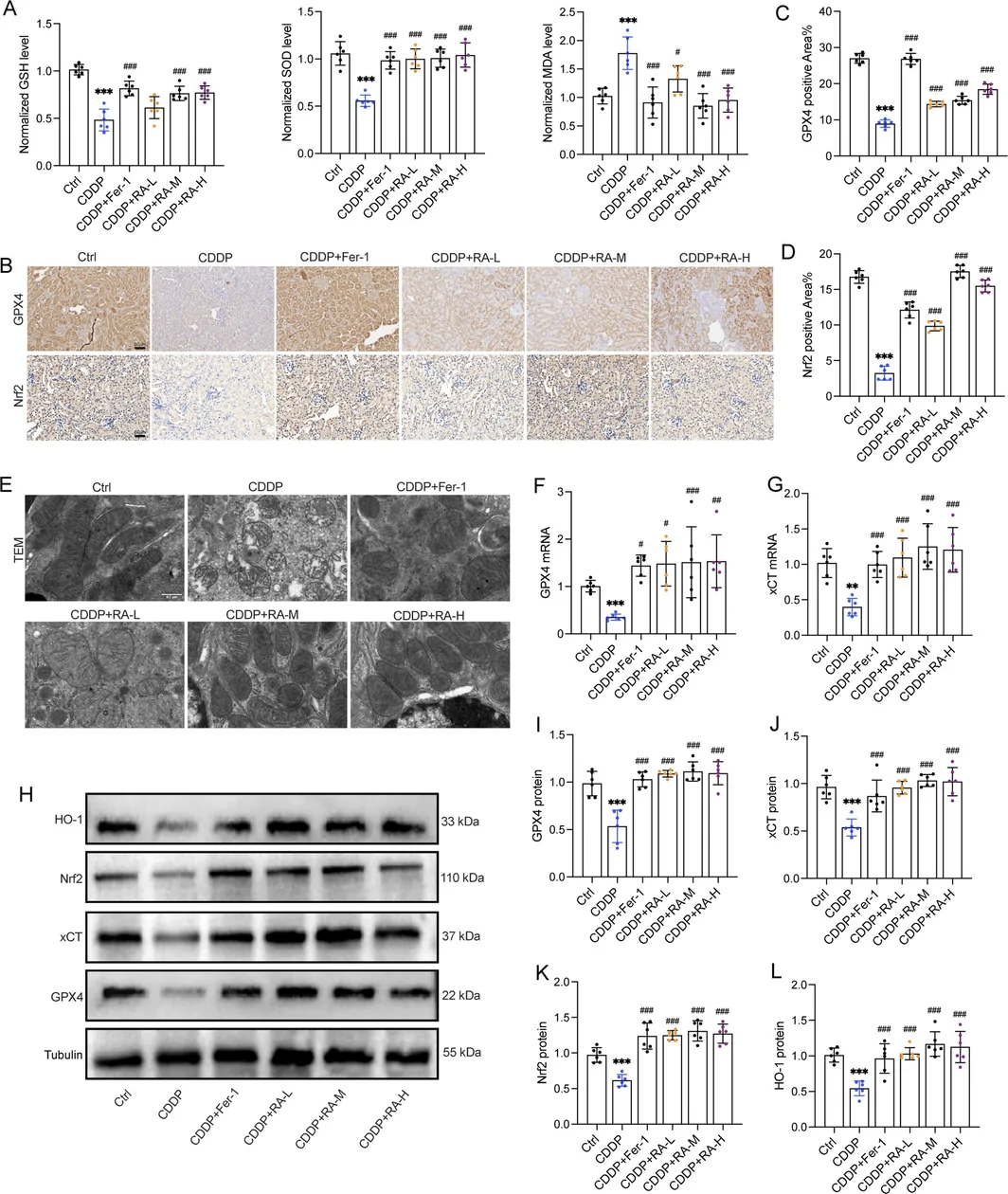
RA suppresses ferroptosis in CDDP‐AKI. (A) GSH, SOD and MDA levels in renal tissues. (B–D) IHC staining show GPX4 and Nrf2 expression in renal tissues with quantitative analysis of positive areas (scale bar = 50 μm, 200× magnification). (E) TEM was used to observe the morphological damage in mitochondria. (F, G) Relative mRNA expression levels of GPX4 and xCT in renal tissues. (H–L) Protein bands and quantitative analysis of GPX4, xCT, Nrf2 and HO‐1 in renal tissues. Six biological replicates were included in each group. Data are presented as mean ± SD. Data were assessed by one‐way ANOVA followed by Tukey's multiple comparisons test. ***p < 0.001, vs. the control group; ^###^ *p* < 0.001, ^##^ *p* < 0.01, ^#^ *p* < 0.05, vs. the CDDP group.

Considering that ferroptosis is involved in CDDP‐AKI pathology, we further investigated whether RA regulated ferroptosis. We first evaluated the anti‐lipid peroxidation capacity of RA. Compared with the control group, the CDDP‐AKI model group showed decreased GSH and SOD levels along with increased MDA levels, all of which were significantly reversed by Fer‐1 or RA (Figure 3A). GPX4 and xCT are key ferroptosis markers. IHC results demonstrated significantly reduced GPX4 protein expression in renal tissues of CDDP‐AKI mice, which was markedly restored after Fer‐1 or RA treatment. Additionally, nuclear expression of Nrf2 was substantially decreased in the CDDP‐AKI group, while both Fer‐1 and RA supplementation enhanced its nuclear translocation (Figure 3B–D). Furthermore, TEM revealed severe mitochondrial damage in the CDDP group, characterized by mitochondrial shrinkage, disrupted cristae, and membrane rupture, which were substantially mitigated by Fer‐1 and RA treatment (Figure 3E). qPCR consistently confirmed that Fer‐1 and RA reversed the CDDP‐induced downregulation of GPX4 and xCT at the mRNA level (Figure 3F,G). WB further demonstrated that CDDP treatment significantly suppressed the protein expression of GPX4, xCT, Nrf2, and HO‐1, and these effects were reversed by Fer‐1 or RA (Figure 3H–L). In conclusion, RA may protect against CDDP‐AKI by regulating ferroptosis through the Nrf2/HO‐1 signaling pathway.

### 3.4 RA Alleviates HK‐2 Cell Injury and Ferroptosis in CDDP‐AKI

**FIGURE 4:**
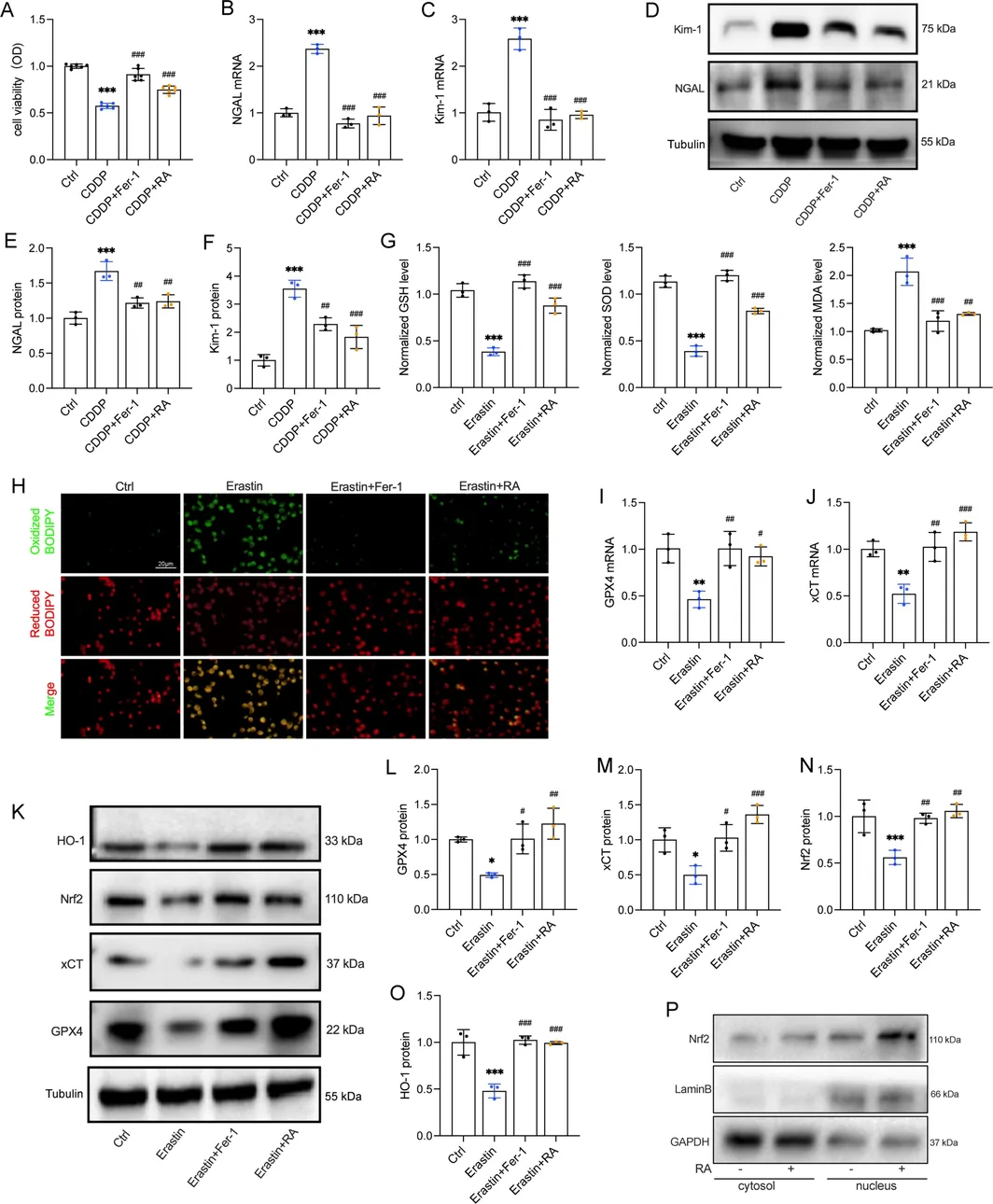
RA protects against HK‐2 cell injury and ferroptosis in CDDP‐AKI. (A) Cell viability was determined by CCK‐8 assay. (B, C) Relative mRNA expression levels of NGAL and Kim‐1 in cells. (D–F) Protein bands and quantitative analysis of NGAL and Kim‐1. (G) GSH, SOD and MDA levels in cells. (H) Representative images of BODIPY‐C11 staining showing intracellular lipid peroxidation levels (scale bar = 20 μm, 200× magnification). (I, J) Relative mRNA expression levels of GPX4 and xCT in cells. (K–O) Protein bands and quantitative analysis of GPX4, xCT, Nrf2 and HO‐1 in cells. (P) Nrf2 protein expression in the cytoplasm and nucleus with or without RA treatment. Six biological replicates for (A) and three biological replicates for (B–P). Data are presented as mean ± SD. Data were assessed by one‐way ANOVA followed by Tukey's multiple comparisons test. ***p < 0.001, **p < 0.01, vs. the control group; ^###^ *p* < 0.001, ^##^ *p* < 0.01, ^#^ *p* < 0.05, vs. the CDDP group.

We further validated the inhibitory effects of RA on renal tubular epithelial cell injury and ferroptosis in vitro. CCK‐8 assays demonstrated that CDDP significantly suppressed HK‐2 cell viability, while RA reversed this inhibitory effect (Figure 4A). qPCR and Western blot analyses showed that RA reduced both mRNA and protein expression levels of NGAL and Kim‐1 (Figure 4B–F). Erastin, a ferroptosis inducer that functionally inhibits the cystine/glutamate antiporter (System Xc^−^) (Dixon et al. 2012), was utilized to establish a ferroptosis model in HK‐2. RA restored the erastin‐induced decreases in intracellular GSH and SOD levels, while reducing MDA content (Figure 4G). Lipid peroxidation was detected using the BODIPY‐C11 fluorescent probe, and the results indicated that RA suppressed erastin‐induced lipid peroxidation, as evidenced by a reduced shift in BODIPY‐C11 fluorescence from red to green (Figure 4H). Furthermore, RA recovered the mRNA expression levels of GPX4 and xCT (Figure 4I,J). WB analysis further demonstrated that RA abolished the erastin‐induced decreases in GPX4, xCT, Nrf2, and HO‐1 protein levels (Figure 4K–O). In addition, we observed that RA promoted the nuclear protein expression of Nrf2, indicating enhanced nuclear translocation (Figure 4P). Together, these findings confirm that RA alleviates CDDP‐induced HK‐2 cell injury and inhibits erastin‐induced ferroptosis through the Nrf2/HO‐1 signaling pathway.

### 3.5 FYN Is Identified as the Direct Target of RA

**FIGURE 5:**
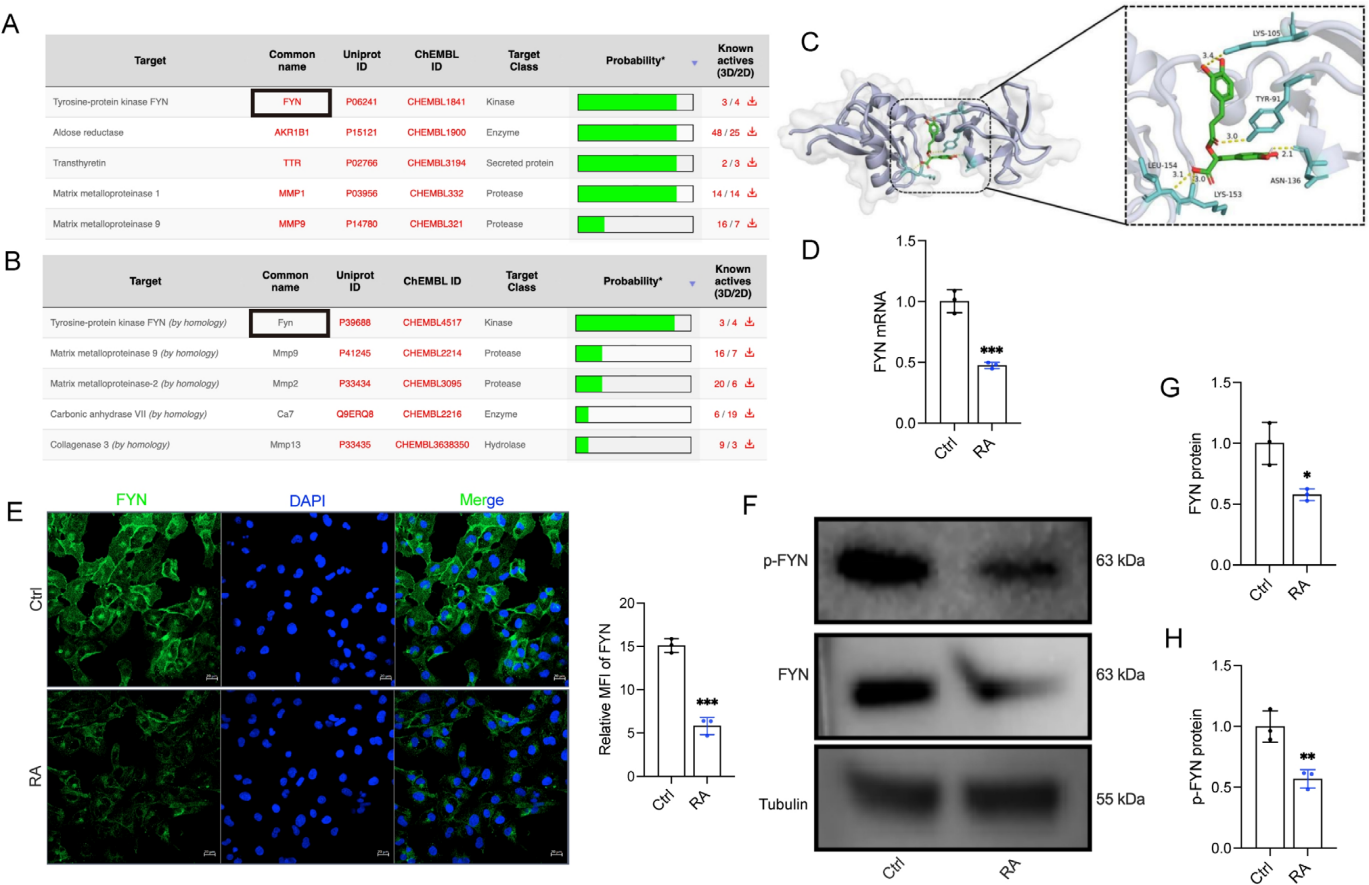
RA interacts with FYN in HK‐2 cells. (A) SwissTargetPrediction of potential RA targets in *Mus musculus* . (B) SwissTargetPrediction of potential RA targets in *Homo sapiens* . (C) Molecular docking model of RA with FYN. (D) Relative mRNA level of FYN. (E) Representative IF images of FYN (scale bar = 20 μm, 200× magnification). (F–H) Protein bands and quantitative analysis of FYN and p‐FYN. Three biological replicates were included in each group. Data are presented as mean ± SD. Comparisons between two groups were performed using the unpaired Student's *t*‐test. ***p < 0.001, **p < 0.01, *p < 0.05, vs. the ontrol group.

To investigate the molecular mechanism by which RA regulates ferroptosis, we first retrieved the chemical structure of RA from the PubChem database and used the SwissTargetPrediction database to forecast its potential therapeutic targets. In *Mus musculus* , the tyrosine‐protein kinase FYN was identified as the most promising target (Figure 5A). Notably, when the species was selected as *Homo sapiens* , SwissTargetPrediction still ranked FYN as the primary candidate target for RA (Figure 5B). Furthermore, molecular docking revealed a direct binding interaction between RA and FYN (Figure 5C). We next examined the effect of RA on FYN expression and its phosphorylation level. RA significantly reduced the mRNA expression level of FYN (Figure 5D). IF showed that FYN was mainly localized in the cell cytoplasm, and RA markedly decreased its fluorescence intensity (Figure 5E). WB further confirmed that RA downregulated FYN protein expression and inhibited its activity (Figure 5F–H).

### 3.6 Pharmacological Inhibition of FYN Improves Renal Damage and Lipid Metabolic Disorders in CDDP‐AKI Mice

**FIGURE 6:**
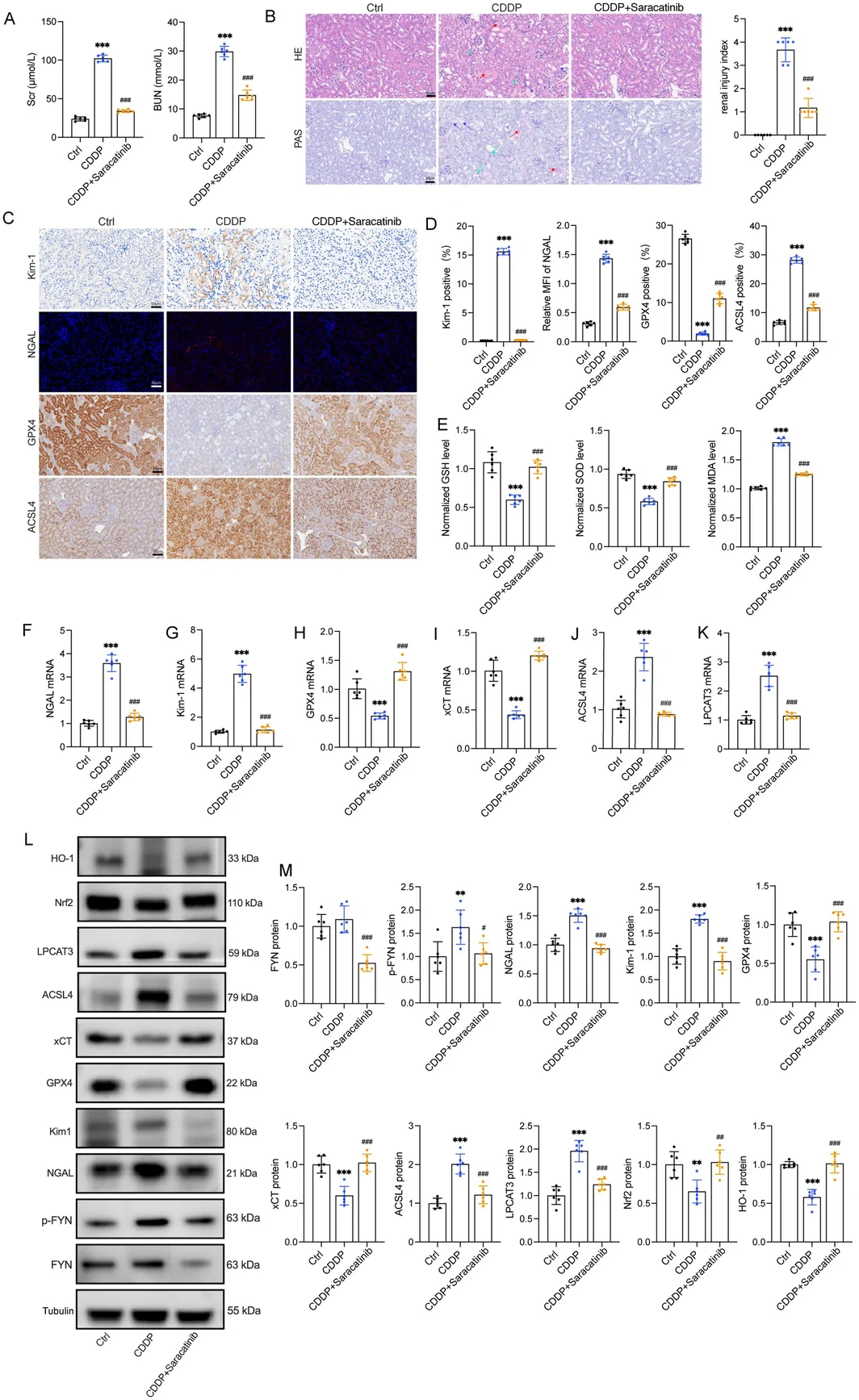
Saracatinib alleviates renal injury and lipid metabolism dysregulation in CDDP‐AKI mice. (A) The levels of Scr and BUN. (B) H&E and PAS staining of renal tissues, and tubular injury scores (scale bar = 50 μm, 200× magnification). Red arrows, renal tubular cast formation; blue arrows, renal tubular dilatation and loss of brush border; green arrows, renal tubular necrosis. (C, D) IHC staining for Kim‐1, GPX4 and ACSL4 and IF staining for NGAL in renal tissues, and quantitative analysis (scale bar = 50 μm, 200× magnification). (E) GSH, SOD and MDA levels in renal tissues. (F–K) Relative mRNA level of NGAL, Kim‐1, GPX4, xCT, ACSL4 and LPCAT3. (L, M) Protein bands and quantitative analysis of FYN, p‐FYN, NGAL, Kim‐1, GPX4, xCT, ACSL4, LPCAT3, Nrf2 and HO‐1. Six biological replicates were included in each group. Data are presented as mean ± SD. Data were assessed by one‐way ANOVA followed by Tukey's multiple comparisons test. ***p < 0.001, **p < 0.01, vs. the control group; ^###^ *p* < 0.001, ^##^ *p* < 0.01, vs. the CDDP group.

To further investigate the role of FYN in CDDP‐AKI, we pharmacologically inhibited FYN by intraperitoneal administration of Saracatinib. We found that pharmacological inhibition of FYN significantly improved renal function in mice with CDDP‐AKI (Figure 6A) and ameliorated renal tubular histopathological injury (Figure 6B). ACSL4 and LPCAT3 are key enzymes involved in lipid composition regulation and ferroptosis promotion. IHC revealed that the expression of Kim‐1, NGAL, and ACSL4 was markedly increased, while GPX4 expression was decreased in CDDP‐AKI mice. These alterations were significantly reversed by Saracatinib treatment (Figure 6C,D). Saracatinib restored GSH and SOD levels and reduced MDA, suggesting the inhibition of lipid peroxidation (Figure 6E). Furthermore, CDDP treatment significantly increased the mRNA levels of Kim‐1, NGAL, ACSL4, and LPCAT3, while decreasing the mRNA levels of GPX4 and xCT, which were markedly reversed by Saracatinib (Figure 6F–K). Western blot analysis demonstrated that Saracatinib significantly reduced the protein expression of NGAL, Kim‐1, ACSL4, LPCAT3, total FYN, and phosphorylated FYN, and upregulated the protein levels of GPX4, xCT, Nrf2, and HO‐1 (Figure 6L,M).

### 3.7 Knockdown of FYN Suppresses CDDP‐Induced HK‐2 Cell Injury and Lipid Metabolism Disorder

**FIGURE 7:**
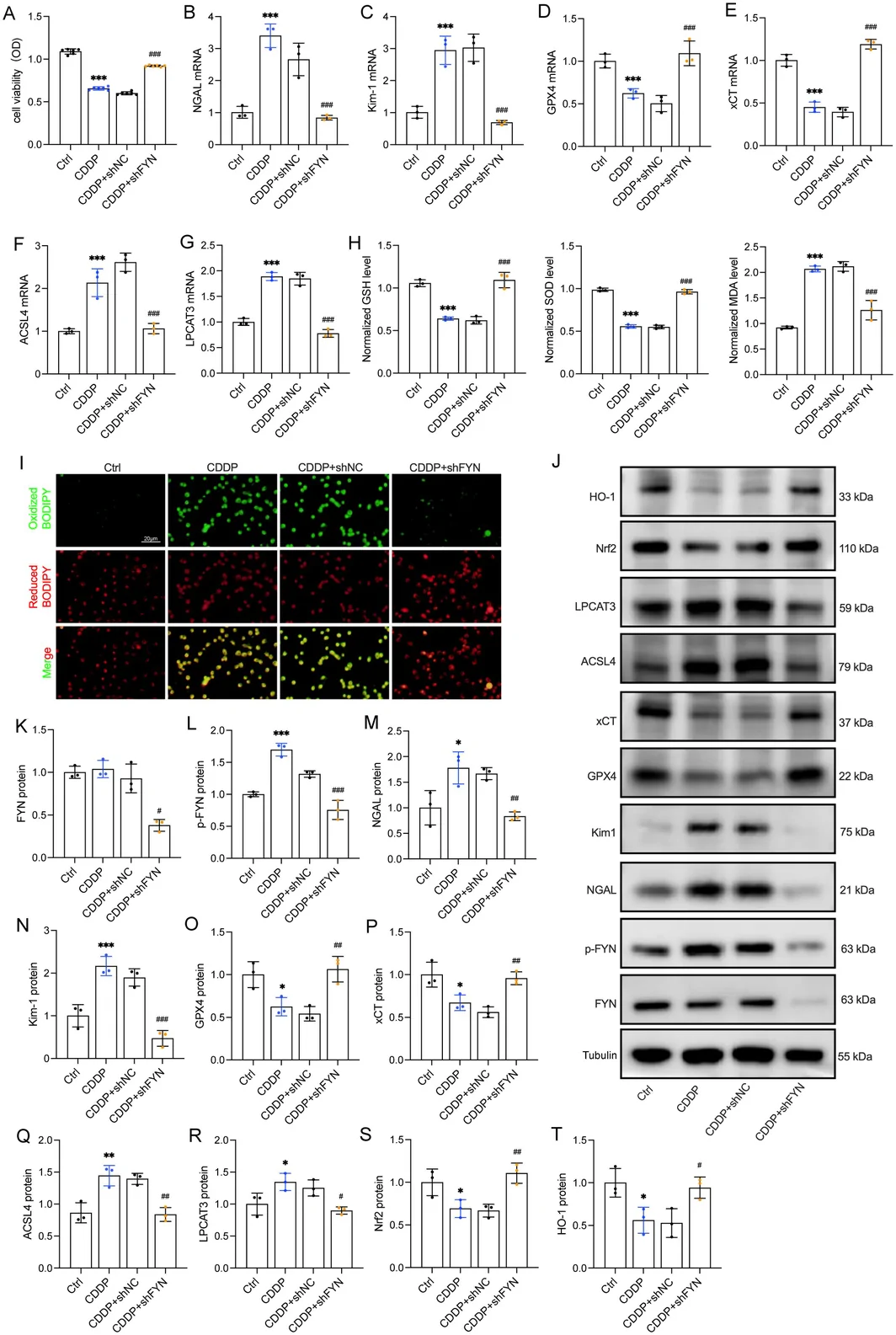
Knockdown of FYN alleviates CDDP‐induced injury and lipid dysregulation in HK‐2 cells. (A) Cell viability assessed by CCK‐8 assay. (B–G) Relative mRNA levels of NGAL, Kim‐1, GPX4, xCT, ACSL4, and LPCAT3 in cells. (H) Levels of GSH, SOD, and MDA in cells. (I) Representative images of BODIPY‐C11 staining showing intracellular lipid peroxidation (scale bar = 20 μm, 200× magnification). (J–T) Protein bands and quantitative analysis of FYN, p‐FYN, NGAL, Kim‐1, GPX4, xCT, ACSL4, LPCAT3, Nrf2, and HO‐1. Three biological replicates were included in each group. Data are presented as mean ± SD. Data were assessed by one‐way ANOVA followed by Tukey's multiple comparisons test. ***p < 0.001, **p < 0.01, *p < 0.05, vs. the control group; ^###^ *p* < 0.001, ^##^ *p* < 0.01, ^#^ *p* < 0.05, vs. the CDDP+shNC group.

To further validate the role of FYN in renal tubular epithelial cell injury and lipid metabolism dysregulation in vitro, we knocked down FYN in HK‐2 cells using shRNA. The CCK‐8 assay indicated that FYN knockdown significantly enhanced the viability of HK‐2 cells (Figure 7A). qPCR analysis showed that FYN silencing downregulated the mRNA levels of NGAL, Kim‐1, ACSL4, and LPCAT3, while increasing the mRNA expression of GPX4 and xCT (Figure 7B–G). Furthermore, FYN knockdown markedly increased cellular levels of GSH and SOD while reducing MDA content (Figure 7H) and significantly suppressed the shift in BODIPY‐C11 fluorescence from red to green in HK‐2 cells, indicating inhibition of lipid peroxidation (Figure 7I). Western blot analysis revealed that FYN knockdown decreased the protein expression of NGAL, Kim‐1, ACSL4, LPCAT3, total FYN, and phosphorylated FYN, while increasing the expression of GPX4, xCT, Nrf2, and HO‐1 (Figure 7J–T). In summary, these results demonstrate that FYN knockdown effectively attenuates CDDP‐induced renal tubular epithelial cell injury and dysregulation of lipid metabolism.

## 4 Discussion

CDDP is a first‐line chemotherapeutic agent for multiple cancers, yet its clinical application is severely restricted by severe side effects, especially nephrotoxicity and ototoxicity (Sung et al. 2024; Gupta et al. 2025). Herein, we investigated the protective effects of natural product RA against CDDP‐AKI. Mechanistically, RA alleviates CDDP‐AKI by targeting FYN to modulate ferroptosis, offering a novel experimental basis for the targeted prevention and treatment of CDDP‐related renal injury.

RA is a natural polyphenol abundant in Lamiaceae and Boraginaceae plants, with potent antioxidant, anti‐inflammatory, and antibacterial activities (Jakovljević et al. 2025). It has been widely reported to treat multiple diseases, including multiple cancers (Zhou et al. 2022; Şengelen and Önay‐Uçar 2024), osteoarthritis (Eo and Kim 2017), neurotoxicity (Wang, He, et al. 2024), and allergic dermatitis (Shim et al. 2024). It blocks ferroptosis through the Nrf2/Keap1 axis to relieve crush syndrome‐related AKI, inhibits the NLRP3 inflammasome, and activates the SIRT1/Nrf2/HO‐1 pathway to improve ciprofloxacin‐triggered renal oxidative damage (Qiao et al. 2024; Hung et al. 2024; Abduh et al. 2023). Single‐cell analyses further confirm that RA mitigates renal tubular injury, suppresses macrophage inflammation and oxidative stress, and weakens natural killer cell cytotoxicity (Chen et al. 2024). Collectively, RA exhibits prominent reno‐protective properties. Consistent with previous evidence, our results demonstrated that RA markedly alleviated tubular damage, inflammatory infiltration, and mitochondrial dysfunction in CDDP‐AKI, highlighting its promising therapeutic value for cisplatin‐induced nephrotoxicity.

Growing evidence links ferroptosis to tissue and cellular damage in various kidney diseases (Sun et al. 2023; Feng et al. 2023). In our study, RA restored GSH and SOD levels, upregulated GPX4 and xCT expression, decreased MDA levels, and effectively suppressed lipid ROS accumulation. Ferroptosis is associated with mitochondrial morphological changes, such as increased membrane density, shrinkage, and reduced/absent cristae (Feng et al. 2022), and TEM showed RA ameliorated such damage in CDDP‐AKI. Under oxidative stress, Nrf2 translocates to the nucleus, initiating transcription of protective genes and promoting GSH synthesis to enhance antioxidant capacity and eliminate lipid peroxides. Here, our findings demonstrate RA activates the Nrf2/HO‐1 pathway to inhibit ferroptosis in CDDP‐AKI.

FYN, a key Src family kinase, is hyperactive in cancers and targeted inhibition of its expression can counteract disease progression (Peng and Fu 2023), and participates in neural function, glucose‐lipid metabolism, and atherosclerosis alleviation (Matrone et al. 2020; Xue et al. 2025). Additionally, FYN is a critical negative modulator of the Nrf2/HO‐1 axis. Activated by phosphorylation, FYN induces Nrf2 phosphorylation at Tyr568, which facilitates Nrf2 nuclear export and degradation, and represses HO‐1 transcription (Lee et al. 2023). We found that RA directly binds FYN and reduces its activity. Saracatinib, an inhibitor of the Src family, suppresses both the expression and activity of FYN (Du et al. 2020). Pharmacological inhibition of FYN alleviates CDDP‐AKI tubular damage and ferroptosis, and represses Nrf2/HO‐1 signaling. These findings indicate that RA may exert its protective effects in CDDP‐AKI by reducing FYN activity. However, a limitation is that specific FYN inhibitors are currently unavailable due to high structural homology among Src family kinases, and further specific FYN knockout in vivo experiments will strengthen the evidence for FYN's role in CDDP‐AKI.

In summary, our data demonstrate that RA alleviates CDDP‐AKI by inhibiting ferroptosis, which is associated with the targeted suppression of FYN activity and subsequent activation of the Nrf2/HO‐1 signaling pathway. This study highlights the protective role of RA in CDDP‐AKI and suggests its potential as a promising candidate for renal protective therapy.

## Author Contributions

**Chan Huang:** writing – original draft. **Ningxun Cui:** validation, investigation. **Aiqing Zhang:** writing – review and editing, conceptualization. **Lu Jiang:** validation, data curation. **Wei Zhou:** writing – review and editing, conceptualization. **Shuang Xu:** data curation. **Gaoting Qu:** formal analysis, methodology. **Huimin Shi:** writing – original draft.

## Funding

The work was supported by the Medical and Health Science Program of Zhejiang Province (2025HY0499, 2025ZR046).

## Conflicts of Interest

The authors declare no conflicts of interest.

## Supporting information


**Table S1:** Primers sequences for qRT‐PCR.
**Table S2:** Antibodies for Western blot.

## Data Availability

The data that support the findings of this study are available from the corresponding author upon reasonable request.
