# Supplementary material for: Rosmarinic Acid Alleviates Cisplatin‐Induced Acute Kidney Injury by Targeting FYN to Regulate Ferroptosis
Source: Food Sci Nutr. 2026 Aug 27;14(9):e72281. doi: 10.1002/fsn3.72281 (PMC13520794; doi:10.1002/fsn3.72281)
Supplement: Supplementary file 1 [file FSN3-14-e72281-s001.docx]

**Supplementary Table 1. Primers sequences for qRT-PCR**

| **Gene** | **Primer sequence 5’ － 3’** |
| --- | --- |
| M - β-actin - F  M - β-actin - R | CATCCGTAAAGACCTCTATGCCAAC  ATGGAGCCACCGATCCACA |
| M - Kim-1 - F  M - Kim-1 - R | CTATGTTGGCATCTGCATCG  AAGGCAACCACGCTTAGAGA |
| M - NGAL - F  M - NGAL - R | GCAGGTGGTACGTTGTGGG  CTCTTGTAGCTCATAGATGGTGC |
| M - GPX4 - F  M - GPX4 - R | ATACGCTGAGTGTGGTTTGC  CTTCATCCACTTCCACAGCG |
| M - xCT - F  M - xCT - R | CCTCTGACGATGGTGATGCTCTTC  GGTGCTGAATGGGTCCGAGTAAAG |
| M - ACSL4 - F  M - ACSL4 – R | GGACTAGGACCGAAGGACACAT  GGAACAGCGGCCATAAGTGT |
| M - LPCAT3 - F  M - LPCAT3 - R | GATGGAGGCAAAGACGGGAA  TCCAGCAATGAAGGGACACC |
| M - IL-6 - F  M - IL-6 - R | ACAAAGCCAGAGTCCTTCAGAGAG  TTGGATGGTCTTGGTCCTTAGCCA |
| M - TNF-α- F  M - TNF-α- R | TCCCCAAAGGGATGAGAAG  CACTTGGTGGTTTGCTACGA |
| M - IL-1β- F  M - IL-1β- R | TGACAGTGATGAGAATGACCTGTTC  TGCTGCTGCGAGATTTGAAG |
| H - β-actin - F  H - β-actin - R | CATGTACGTTGCTATCCAGGC  CTCCTTAATGTCACGCACGAT |
| H - Kim-1 - F  H - Kim-1 - R | CCAGGCAGAACCATGAACCA  CTGTCACGGTGTCATTCCCA |
| H - NGAL - F  H - NGAL - R | ATTCTGCTGTCCCCATCTCG  CCAGCTCCCTCAATGGTGTT |
| H - GPX4 - F  H - GPX4 - R | GAGGCAAGACCGAAGTAAACTAC  CCGAACTGGTTACACGGGAA |
| H - xCT - F  H - xCT - R | AGGGATTGGCTTCGTCATCA  GGCAGATTGCCAAGATCTCAA |
| H - ACSL4 - F  H - ACSL4 – R | TGTGGACAATAAGGCTATCA  TGGTCTACTTGGAGGAATG |
| H - LPCAT3 - F  H - LPCAT3 - R | AGCCCTGATACCTTCTCTGATGG  GCATGTAGCCTGGAACTGTGTAG |
| H - FYN - F  H - FYN - R | TCCTTCTCCTTTCAGTCAGGAAT  CTTACATTGCACACAGCCCA |

**Supplementary Table 2. Antibodies for Western blot**

| **Gene** | **Corporation** | **Catalog** |
| --- | --- | --- |
| Tubulin | Affinity | AF-7011 |
| Kim-1 | R&D Systems | AF-1817 |
| NGAL | Affinity | DF-6816 |
| GPX4 | HUABIO | ET-170645 |
| xCT | Affinity | DF12509 |
| FYN | HUABIO | HA721577 |
| p-FYN | Affinity | AF3102 |
| ACSL4 | HUABIO | ET7111-43 |
| LPCAT3 | Proteintech | 67882-1 |
| Nrf2 | Affinity | AF-7006 |
| HO-1 | Affinity | AF-5393 |
| GAPDH | Affinity | AF-7021 |
| LaminB1 | Affinity | AF-5161 |
| Goat anti-mouse IgG | Abbkine | A21010 |
| Goat anti-rabbit IgG | Abbkine | A21030 |
